# Hypersensitive quantification of major astringency markers in food and wine by substoichiometric quenching of silicon-rhodamine conjugates

**DOI:** 10.1016/j.fochx.2024.101592

**Published:** 2024-06-25

**Authors:** Daniel Bátora, Ágnes Dienes-Nagy, Liming Zeng, Christian E. Gerber, Jérôme P. Fischer, Martin Lochner, Jürg Gertsch

**Affiliations:** a Institute of Biochemistry and Molecular Medicine, University of Bern, 3012 Bern, Switzerland; b Graduate School for Cellular and Biomedical Sciences, University of Bern, 3012 Bern, Switzerland; c Agroscope Changins, 1260 Nyon, Switzerland; d University of Applied Sciences and Arts of Western Switzerland (HES-SO), Changins Viticulture and Enology College, 1260 Nyon, Switzerland

## Abstract

Tannins are chemically diverse polyphenols in plant-derived products that not only show diverse biological activities but also play a crucial role in determining the sensory attributes of food and beverages. Therefore, their accurate and cost-effective quantification is essential.

Here, we identified a novel fluorescence quenching mechanism of different synthetic rhodamine fluorophores, with a high selectivity towards tannic acid (TA) and catechin-3-gallate (C3G) compared to a structurally diverse panel of tannins and polyphenols. Specific chemical conjugates of silicon-rhodamine with alkyl linkers attached to bulky apolar moieties had a limit of detection near 500 pM and a linear range spanning 5–100 nM for TA. We validated the assay on 18 distinct red wine samples, which showed high linearity (R^2^ = 0.92) with methylcellulose precipitation with no interference from anthocyanins. In conclusion, a novel assay was developed and validated that allows the sensitive and selective quantification of major astringency markers abundant in food and beverages.

## Highlights

• First evidence of quenching of silicon-rhodamines (SiR) by tannins
• Tannins quench specific SiR conjugates with differential potency
• Tannic acid had a detection limit of 500 pM and a linear range of 5–100 nM
• Selectivity was evaluated on a diverse panel of phenolic compounds.
• The assay was validated in diverse red wine samples

## 1 Introduction

Tannins are structurally diverse polyphenolic natural products that are widely distributed in plants and fruits (Chung et al., 1998). Generally, the term tannin indicates the strong complexing capability with macromolecules like proteins of different polyphenols that contain multiple hydroxyl groups in sterically accessible positions (Zhang et al., 2023). On a structural level, such complexing capability is reported in hydrolysable tannins (Das et al., 2020), galloyl esters of flavan-3-ol monomers (Hara et al., 2012) and condensed tannins which are oligo- and polymers of flavan-3-ols (Panzella & Napolitano, 2022).

Hydrolysable tannins such as tannic acid (TA), which exhibit a starlike structure decorated by hydroxyl groups, are utilized in different technologies in the medical, chemical and food industries owing to their highly diverse chemical and biological activities (Bigham et al., 2022; Pavez et al., 2022; Soyocak et al., 2019). While TA primarily possesses beneficial properties for health, the molecule can also pose health and environmental hazards when consumed in excessive amounts (Nghia et al., 2023). In pharmaceutical research, TA has demonstrated antimicrobial (Farha et al., 2020), antioxidant (Andrade et al., 2006) and adjuvant (Cabral-Hipólito et al., 2022) properties. On the other hand, high TA intake has been associated to adverse effects such as vomiting, abdominal pain, unpleasant taste (Nghia et al., 2023), and can limit the absorption of essential minerals such as iron; and amino acids by forming irreversible tannin-metal and tannin-protein complexes (Samtiya et al., 2020). Furthermore, in micromolar concentrations, TA and related compounds are pan-assay interference compounds (PAINS) (Dahlin et al., 2021). Thus, the quantification of TA and structurally related tannins is relevant for the quality control, the evaluation of potential health and environmental impact in foodstuff and the assessment of sensory characteristics (Pavez et al., 2022), stability (Liao et al., 2023) and shelf-life (Ma et al., 2021).

Condensed tannins are naturally abundant in plant derived food and beverages and are the key ingredients that determine the sensory attributes of wine. In wine, the structure and quantity of condensed tannins depend on the grape variety (Harbertson et al., 2008), the climate and the soil (Rouxinol et al., 2023), the viticultural and enological practices (Picariello et al., 2020), and the wine-aging process (Tu et al., 2022). Therefore, a chemical and sensorial assessment, and its association with the content of condensed tannins is essential in winemaking to ensure a satisfactory sensory experience.

It is worth noting that in the food industry, tannin content is generally quantified as a class rather than individual molecules and assays use either specific markers or nonspecific reactions as proxy for the sensory attributes (Sarneckis et al., 2006). Among the traditional tannin quantification techniques, chromatographic and mass spectrometric methods provide high accuracy (Degano et al., 2019), but are sophisticated, require expensive equipment and complicated operating procedures, and are unsuitable for large-scale on-site analysis. The adaptation of new colorimetric approaches (Chen et al., 2013) using nanoparticles are hindered by low selectivity and require time-consuming and multi-step sample processing due to high sample absorbance (Li et al., 2015). Assays based on fluorescence or chemiluminescence may offer an alternative to traditional methods with high sensitivity and with minimal sample processing interference (Wang et al., 2024). For example, a recently developed quantification method for TA with a λ_em_ of 510 nm (IC_50_ = 8 μM) based on fluorescence-quenching of Eu^3+^/polyethyleneimine complex reported a detection range of 160 to 66,000 nM of TA and demonstrated adaptability to on-site analysis with a smartphone device (Nghia et al., 2023).

However, the absence of a) a comprehensive characterization of the selectivity of new methods towards structurally different tannins and b) an extensive validation on real-word samples compared to reference methods might limit their application in practice.

Herein, we present a novel, single-component tannin quantification assay using silicon-rhodamine (SiR) derivatives which were most selective towards TA and (−)-catechin-3-gallate (C3G) showing superior quenching to rhodamine fluorescence (Wang et al., 2024). We discovered that the potency of the quenching reaction was highly sensitive to chemical moieties conjugated to the rhodamine fluorophore resulting in tunable sensitivities in the range of 5 nM to 1000 nM. A comprehensive selectivity screening revealed no interference from malvidins and ellagitannins. The method was validated by comparison with the methylcellulose method in 18 red wine samples from five grape varieties, to which it showed a strong linearity (R^2^ = 0.92) and required 150 times less sample volume.

## 2 Materials and methods

### 2.1 Synthesis procedures

#### 2.1.1 General remarks

Reagents and organic solvents were purchased from commercial suppliers and used without further purification. Deionized water produced in house or commercially available Milli-Q® (mQ) water was used depending on the application. Aqueous solutions of sodium hydroxide, hydrogen chloride, saturated ammonium chloride, saturated sodium chloride (brine) were prepared with deionized water. High performance liquid chromatography (HPLC) was performed using a Thermo Fisher Scientific UltiMate 3000 RSLCnano System composed of a DIONEX UltiMate 3000 Pump, a DIONEX UltiMate 3000 Sampler, a DIONEX UltiMate 3000 Column Compartment and a DIONEX UltiMate 3000 Diode Array Detector. The measurements were conducted using acetonitrile (+ 0.1% trifluoroacetic acid) and mQ water (+ 0.1% trifluoroacetic acid) as eluents and an Acclaim™ 120 C18 column (Thermo Scientific™). Thin layer chromatography (TLC) was performed using Macherey-Nagel ALUGRAM® Xtra SIL G/UV_254_ plates coated with 0.20 mm silica gel 60 containing fluorescent indicator. Flash column chromatography (LC) was performed using the Teledyne Isco Combi*Flash©Rf* + system. Teledyne Isco Redi*Sep*©*Rf* dry load cartridges were used for the preparation of dry loads on silica gel. Teledyne Isco Silica Redi*Sep*©*Rf* prepacked silica flash columns of three sizes (12 g, 40 g, 120 g) were used. Nuclear magnetic resonance spectroscopy (NMR) was performed at the Department of Chemistry, Biochemistry and Pharmaceutical Sciences, Universität Bern (Furrer Group) using a Bruker AVANCE III HD 300 GA spectrometer with a magnetic field of 7.05 Tesla and operating frequencies of 300.13 MHz for ^1^H measurements and 75.48 MHz for ^13^C measurements. High resolution mass spectrometry (HRMS) was performed by the mass spectrometry service (Schürch group) at the Department of Chemistry, Biochemistry and Pharmaceutical Sciences, Universität Bern. The measurements were performed using electrospray ionization (ESI) and a ThermoScientific LTQ Orbitrap XL mass spectrometer with high mass resolution (m/Δm > 100′000) and accuracy (Δm < 3 ppm). The previously published synthetic procedure (Ozhathil et al., 2018) to generate similar anthranilic anilide compounds was slightly adapted for the synthesis of methyl 4-chloro-2-(2-chloroacetamido) benzoate, 4-chloro-2-(2-(2-(prop-2-yn-1-yloxy)phenoxy)acetamido)benzoate and 4-chloro-2-(2-(2-(prop-2-yn-1-yloxy)phenoxy)acetamido)benzoic acid. The silicon rhodamine dye (*N*-(7-(dimethylamino)-10-(4-(((2,5-dioxopyrrolidin-1-yl)oxy)carbonyl)-2-methylphenyl)-5,5-dimethyldibenzo[*b,e*]silin-3(5*H*)-ylidene)-*N*-methylmethanaminium chloride) and the alkane linker (6-azidohexan-1-amine) were both synthesized according to a previously published synthetic procedure (Grossenbacher et al., 2022).

**Fig. 1:**
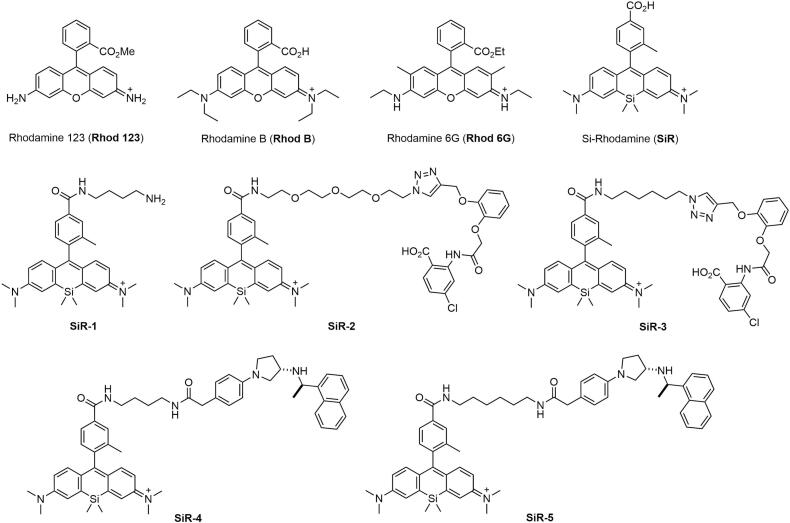
Chemical structure of rhodamine fluorophores and chemical conjugates of silicon-rhodamine (SiR) used in the current study.

The synthesis, mass spectrometry and NMR data for SiR-1, SiR-4 and SiR-5 are described in detail in our previous report (Bátora et al., 2024). The syntheses of SiR-2 and SiR-3 and their intermediates are described below and are depicted in Scheme S1. The chemical structures of the SiR-conjugates and the rhodamine fluorophores are shown in Fig. 1.

#### 2.1.2 Synthesis of 2-(prop-2-yn-1-yloxy)phenol

Pyrocatechol (3.33 g, 30.24 mmol) was dissolved in dry acetone (150 mL) and potassium carbonate (2.29 g, 16.63 mmol, 0.55 eq.) was added. The reaction vessel was put under an argon atmosphere and the mixture was stirred at 21 °C for 1 h. Propargyl bromide (3.60 g, 30.24 mmol, 1 eq.) in toluene (80 wt%) was slowly added *via* syringe for 35 min. The reaction mixture was then stirred at 65 °C and monitored by thin layer chromatography (eluent: cyclohexane / ethyl acetate, 4:1). After satisfactory conversion of the starting material into the product and side products, the reaction mixture was cooled down to room temperature and the solvents were evaporated *in vacuo*. Water and aqueous hydrogen chloride (2 M) was added, and the mixture was stirred at 21 °C for 10 min. The product was then extracted with dichloromethane and the combined organic phases were washed with brine containing aqueous hydrogen chloride, dried over magnesium sulphate, filtered through cotton and the volatiles were evaporated under reduced pressure. The crude solid product was purified by flash column chromatography (eluent: cyclohexane / ethyl acetate, gradient from 0% to 20% ethyl acetate) and dried *in vacuo*. **Yield** 26%, 1.17 g, 7.90 mmol. ^**1**^**H NMR** (300 MHz, DMSO‑*d*_6_) *δ* 9.07 (s, 1H), 6.98 (d, *J* = 7.6 Hz, 1H), 6.84–6.78 (m, 2H), 6.78–6.68 (m, 1H), 4.75 (d, *J* = 2.4 Hz, 2H), 3.53 (t, *J* = 2.4 Hz, 1H). ^**13**^**C NMR** (75 MHz, DMSO) *δ* 147.20, 145.40, 122.05, 118.97, 116.06, 115.07, 79.64, 78.06, 56.14. **HRMS** (ESI) *m/z* calculated for [M-H]^−^ 147.0452, found 147.0455.

#### 2.1.3 Synthesis of methyl 4-chloro-2-(2-chloroacetamido)benzoate

Methyl 2-amino-4-chlorobenzoate (1.7876 g, 9.6308 mmol) and potassium carbonate (2.6620 g, 19.2616 mmol, 2 eq.) were dissolved in tetrahydrofuran (150 mL) and stirred for 10 min at room temperature. Not all potassium carbonate dissolved completely. The mixture was then cooled in an ice bath and chloroacetyl chloride (1.0919 g, 9.6676 mmol, 0.77 mL, 1 eq.) was added dropwise *via* syringe. This mixture was stirred at 0 °C for 10 min and further stirred at room temperature for 16 h. The reaction mixture showed a pale pink color and was monitored by thin layer chromatography (eluent: cyclohexane / ethyl acetate, 4:1). After full conversion of the starting material, water was added to the reaction mixture and the product was extracted with ethyl acetate. The combined organic phases were then washed with brine, dried over magnesium sulphate, filtered through celite and the volatiles were evaporated under reduced pressure. The crude pale-yellow and solid product was purified by flash column chromatography (eluent: cyclohexane / ethyl acetate, gradient from 0% to 20% ethyl acetate). The product (white powder) was dried *in vacuo*. **Yield** quant., 2.5 g, 9.5387 mmol. ^**1**^**H NMR** (300 MHz, DMSO‑*d*_6_) *δ* 11.44 (s, 1H), 8.52 (d, *J* = 2.2 Hz, 1H), 8.00 (d, *J* = 8.6 Hz, 1H), 7.33 (dd, *J* = 8.6, 2.2 Hz, 1H), 4.48 (s, 2H), 3.89 (s, 3H). ^**13**^**C NMR** (75 MHz, DMSO‑*d*_6_) *δ* 166.75, 165.67, 140.29, 138.75, 132.51, 123.77, 119.84, 115.57, 52.82, 43.35. **HRMS** (ESI) *m/z* [M + H]^+^ calculated for C_10_H_9_Cl_2_NO_3_ 262.0032, found 262.0037.

#### 2.1.4 Synthesis of methyl 4-chloro-2-(2-(2-(prop-2-yn-1-yloxy)phenoxy)acetamido)benzoate

Methyl 4-chloro-2-(2-chloroacetamido) benzoate (0.6 g, 2.29 mmol) was dissolved in dimethylformamide (2 mL) and potassium carbonate (0.63 g, 4.59 mmol, 2 eq.) was added. This mixture was stirred for 10 min at 21 °C (room temperature). Not all potassium carbonate dissolved and remained as a white solid in the flask. 2-(prop-2-yn-1-yloxy)phenol (0.37 g, 2.52 mmol, 1.1 eq.) was then added as a solid and the mixture was stirred at 80 °C. The color of the reaction mixture turned into a deep brown. The reaction was monitored by thin layer chromatography (eluent: cyclohexane / ethyl acetate, 4:1). After full conversion of the starting material, water was added to the mixture resulting in a brown suspension. The aqueous phase was extracted with ethyl acetate and the combined organic phases were then washed with brine, dried over magnesium sulphate, filtered through cotton and the volatiles were evaporated under reduced pressure. The crude product was purified by flash column chromatography (eluent: cyclohexane / ethyl acetate, gradient from 0% to 20% ethyl acetate). **Yield** 59%, 0.50 g, 1.34 mmol. ^**1**^**H NMR** (300 MHz, DMSO‑*d*_6_) *δ* 11.64 (s, 1H), 8.72 (d, *J* = 2.2 Hz, 1H), 8.00 (d, *J* = 8.6 Hz, 1H), 7.30 (dd, *J* = 8.6, 2.2 Hz, 1H), 7.13 (ddd, *J* = 9.3, 7.8, 1.8 Hz, 2H), 6.99 (dtd, *J* = 20.8, 7.5, 1.7 Hz, 2H), 4.86 (d, *J* = 2.4 Hz, 2H), 4.73 (s, 2H), 3.87 (s, 3H), 3.53 (t, *J* = 2.4 Hz, 1H). ^**13**^**C NMR** (75 MHz, DMSO) *δ* 167.94, 166.50, 147.47, 147.27, 140.53, 138.81, 132.53, 123.33, 122.74, 121.84, 119.61, 115.98, 115.05, 114.85, 79.30, 78.21, 69.07, 56.31, 52.68. **HRMS** (ESI) *m/z* calculated for [M + H]^+^ 374.0790, found 374.0790.

#### 2.1.5 Synthesis of 4-chloro-2-(2-(2-(prop-2-yn-1-yloxy)phenoxy)acetamido)benzoic acid

4-chloro-2-(2-(2-(prop-2-yn-1-yloxy)phenoxy)acetamido)benzoate (0.49 g, 1.30 mmol, 1 eq.) was dissolved in methanol (50 mL) and potassium hydroxide (0.22 g, 3.92 mmol, 3 eq.) in water (17 mL) was added later. The reaction mixture was then stirred at 65 °C and monitored by thin layer chromatography (eluents: cyclohexane / ethyl acetate, 4:1 and dichloromethane / methanol, 9:1). After full conversion of the starting material, the mixture was cooled down in an ice bath and aqueous hydrogen chloride (2.7 M) was then added to the reaction mixture. The addition resulted in an immediate precipitation of the product. The suspension was filtered, and the filter cake was washed with aqueous hydrogen chloride (2.7 M) and water. The solid product was dried *in vacuo*. **Yield** quant., 0.580 g, 1.612 mmol. ^**1**^**H NMR** (300 MHz, DMSO‑*d*_6_) *δ* 12.91 (s, 1H), 8.72 (d, *J* = 2.2 Hz, 1H), 8.01 (d, *J* = 8.5 Hz, 1H), 7.20 (dd, *J* = 8.5, 2.2 Hz, 1H), 7.12 (dt, *J* = 7.6, 1.8 Hz, 2H), 6.97 (dtd, *J* = 18.1, 7.5, 1.8 Hz, 2H), 4.85 (d, *J* = 2.4 Hz, 2H), 4.70 (s, 2H), 3.54 (t, *J* = 2.4 Hz, 1H). ^**13**^**C NMR** (75 MHz, DMSO) *δ* 168.33, 167.93, 147.82, 147.56, 141.08, 137.13, 132.88, 122.63, 122.03, 118.80, 117.01, 116.33, 115.63, 79.45, 78.31, 69.52, 56.62. **HRMS** (ESI) *m/z* calculated for [M-H]^−^ 358.0488, found 358.0491.

#### 2.1.6 Synthesis of 2-(2-(2-((1-(2-(2-(2-(2-aminoethoxy)ethoxy)ethoxy)ethyl)-1*H*-1,2,3-triazol-4-yl)methoxy)phenoxy)acetamido)-4-chlorobenzoic acid

2-(2-(2-(2-azidoethoxy)ethoxy)ethoxy)ethan-1-amine (0.14 g, 0.663 mmol, 2 eq.), sodium ascorbate (7.29 mg, 0.037 mmol, 0.1 eq.) and copper(*II*)sulphate pentahydrate (3.3 mg, 0.013 mmol, 0.04 eq.) were dissolved in *tert*-butanol (4 mL) and water (4 mL). The resulting solution was stirred at 25 °C (room temperature) for 10 min. 2-(2-(2-((1-(2-(2-(2-(2-aminoethoxy)ethoxy)ethoxy)ethyl)-1*H*-1,2,3-triazol-4-yl)methoxy)phenoxy)acetamido)-4-chlorobenzoic acid (0.12 g, 0.322 mmol, 1 eq.) was then added as a solid and the mixture was stirred at room temperature. The reaction was monitored by HPLC (eluent: water (+ 0.1% trifluoroacetic acid) / acetonitrile (+ 0.1% trifluoroacetic acid)). After 72 h, full conversion of the starting material was observed, and the solvents were evaporated under reduced pressure. The crude product was purified by reversed phase flash column chromatography (eluent: water (+ 0.1% trifluoroacetic acid) / acetonitrile (+ 0.1% trifluoroacetic acid), gradient from 0% to 100% acetonitrile). The solvents were evaporated under reduced pressure and the purified product was dried *in vacuo*. **Yield** 57%, 0.13 g, 0.190 mmol. ^**1**^**H NMR** (300 MHz, DMSO‑*d*_6_) *δ* 13.95 (s, 1H), 12.06 (s, 1H), 8.74 (d, *J* = 2.2 Hz, 1H), 8.15 (s, 1H), 8.02 (d, *J* = 8.6 Hz, 1H), 7.77 (s, 3H), 7.27 (dd, *J* = 8.6, 2.2 Hz, 1H), 7.23 (dd, *J* = 8.1, 1.6 Hz, 1H), 7.11 (dd, *J* = 7.8, 1.7 Hz, 1H), 7.02 (td, *J* = 7.7, 1.7 Hz, 1H), 6.92 (td, *J* = 7.7, 1.6 Hz, 1H), 5.21 (s, 2H), 4.70 (s, 2H), 4.50 (t, *J* = 5.2 Hz, 2H), 3.80 (t, *J* = 5.2 Hz, 2H), 3.59–3.43 (m, 11H), 2.95 (q, *J* = 5.5 Hz, 2H). ^**13**^**C NMR** (75 MHz, DMSO‑*d*_6_) *δ* 168.46, 148.63, 147.46, 142.68, 141.08, 138.51, 132.97, 124.79, 123.15, 123.05, 121.45, 119.12, 117.10, 117.03, 116.86, 115.20, 69.75, 69.64, 69.58, 69.55, 69.52, 68.68, 66.65, 62.20, 54.92, 49.41. **HRMS** (ESI) *m/z* calculated for [M-H]^−^ 576.1867, found 576.1870.

#### 2.1.7 Synthesis of 2-(2-(2-((1-(6-aminohexyl)-1*H*-1,2,3-triazol-4-yl)methoxy)phenoxy)acetamido)-4-chlorobenzoic acid

6-Azidohexan-1-amine (0.08 g, 0.54 mmol, 1 eq.), sodium ascorbate (8.35 mg, 0.04 mmol, 0.1 eq.) and copper(*II*)sulphate pentahydrate (2.68 mg, 0.01 mmol, 0.02 eq.) were dissolved in *tert*-butanol (12 mL) and water (12 mL). The resulting solution was stirred at 25 °C (room temperature) for 10 min. 4-chloro-2-(2-(2-(prop-2-yn-1-yloxy)phenoxy)acetamido)benzoic acid (0.15 g, 0.42 mmol, 0.8 eq.) was then added as a solid and the mixture was stirred at room temperature. The reaction was monitored by thin layer chromatography (dichloromethane / methanol, 9:1). After 110 h, full conversion of the starting material was observed, and the solvents were evaporated under reduced pressure. The crude product was purified by flash column chromatography (eluent: dichloromethane / methanol, gradient from 0% to 60% methanol) and the volatiles were evaporated under reduced pressure. The purified product was dried *in vacuo*. **Yield** 65%, 0.14 g, 0.27 mmol. ^**1**^**H NMR** (300 MHz, DMSO‑*d*_6_) *δ* 14.47 (s, 1H), 8.63 (d, *J* = 2.2 Hz, 1H), 8.47 (s, 1H), 8.14 (s, 2H), 8.00 (d, *J* = 8.4 Hz, 1H), 7.17–7.03 (m, 3H), 6.92 (dtd, *J* = 18.3, 7.5, 1.7 Hz, 2H), 5.22 (s, 2H), 4.62 (s, 2H), 4.33 (t, *J* = 6.6 Hz, 2H), 2.70 (t, *J* = 7.4 Hz, 2H), 1.74 (p, *J* = 6.9 Hz, 2H), 1.48 (p, *J* = 7.3 Hz, 2H), 1.25 (p, *J* = 7.9 Hz, 2H), 1.11 (p, *J* = 7.0 Hz, 2H). ^**13**^**C NMR** (75 MHz, DMSO) *δ* 167.69, 148.24, 148.14, 143.13, 140.84, 134.26, 132.81, 124.70, 122.54, 121.74, 121.58, 118.15, 116.31, 115.38, 69.93, 62.70, 48.99, 48.60, 38.43, 29.09, 26.60, 24.84, 24.78. **HRMS** (ESI) *m/z* calculated for [M + H]^+^ 502.1852, found 502.1838.

#### 2.1.8 Synthesis of *N*-(10-(4-((2-(2-(2-(2-(4-((2-(2-((2-carboxy-5-chlorophenyl)amino)-2-oxoethoxy)phenoxy)methyl)-1*H*-1,2,3-triazol-1-yl)ethoxy)ethoxy)ethoxy)ethyl)carbamoyl)-2-methylphenyl)-7-(dimethylamino)-5,5-dimethyldibenzo[*b*,*e*]silin-3(5*H*)-ylidene)-*N*-methylmethanaminium chloride (SiR-2)

*N*-(7-(Dimethylamino)-10-(4-(((2,5-dioxopyrrolidin-1-yl)oxy)carbonyl)-2-methylphenyl)-5,5-dimethyldibenzo[*b,e*]silin-3(5*H*)-ylidene)-*N*-methylmethanaminium chloride (25.9 mg, 0.045 mmol, 1.4 eq.) and 2-(2-(2-((1-(2-(2-(2-(2-aminoethoxy)ethoxy)ethoxy)ethyl)-1*H*-1,2,3-triazol-4-yl)methoxy)phenoxy)acetamido)-4-chlorobenzoic acid (22.44 mg, 0.032 mmol, 1 eq.) were dissolved in acetonitrile (3 mL) and stirred at 21 °C (room temperature) for 10 min. The reaction flask was put under an argon atmosphere and *N*,*N*-diisopropylethylamine (10.18 mg, 0.079 mmol, 2.4 eq.) was slowly added to the reaction mixture *via* syringe. The mixture was then protected from light and stirred at room temperature. The reaction was monitored by thin layer chromatography (eluent: dichloromethane / methanol, 9:1) and HPLC (eluent: water (+ 0.1% trifluoroacetic acid) / acetonitrile (+ 0.1% trifluoroacetic acid), gradient from 40% to 95% acetonitrile (+ 0.1% trifluoroacetic acid)). The volatiles were evaporated under reduced pressure after full conversion of the starting material. The crude product was purified by reversed phase flash column chromatography (water +0.1% TFA / acetonitrile +0.1% TFA, gradient from 20% to 60% acetonitrile (+ 0.1% trifluoroacetic acid)) and normal phase flash column chromatography (eluent: dichloromethane / methanol, gradient from 0% methanol to 100% methanol). The purified solid product was dried *in vacuo*. **Yield** 68%, 29.63 mg, 0.039 mmol. ^**1**^**H NMR** (300 MHz, DMSO‑*d*_6_) *δ* 15.14 (s, 1H), 8.81 (t, *J* = 5.5 Hz, 1H), 8.65 (s, 1H), 8.59 (d, *J* = 2.2 Hz, 1H), 8.02–7.92 (m, 2H), 7.88 (d, *J* = 7.9 Hz, 1H), 7.43 (d, *J* = 2.6 Hz, 2H), 7.29–7.14 (m, 2H), 7.08 (ddd, *J* = 7.4, 5.5, 1.9 Hz, 2H), 6.98 (dd, *J* = 8.3, 2.2 Hz, 1H), 6.95–6.73 (m, 6H), 5.20 (s, 2H), 4.59 (s, 2H), 4.53 (t, *J* = 5.3 Hz, 2H), 3.81 (t, *J* = 5.2 Hz, 2H), 3.50 (m, 12H), 3.29 (s, 12H), 1.98 (s, 3H), 0.59 (d, *J* = 7.6 Hz, 6H). **HRMS** (ESI) *m/z* calculated for [M]^+^ 1002.3994, found 1002.3964.

#### 2.1.9 Synthesis of *N*-(10-(4-((6-(4-((2-(2-((2-carboxy-5-chlorophenyl)amino)-2-oxoethoxy)phenoxy)methyl)-1*H*-1,2,3-triazol-1-yl)hexyl)carbamoyl)-2-methylphenyl)-7-(dimethylamino)-5,5-dimethyldibenzo[*b*,*e*]silin-3(5*H*)-ylidene)-*N*-methylmethanaminium chloride (SiR-3)

*N*-(7-(Dimethylamino)-10-(4-(((2,5-dioxopyrrolidin-1-yl)oxy)carbonyl)-2-methylphenyl)-5,5-dimethyldibenzo[*b,e*]silin-3(5*H*)-ylidene)-*N*-methylmethanaminium chloride (0.06 g, 0.10 mmol, 1 eq.) and 2-(2-(2-((1-(6-aminohexyl)-1*H*-1,2,3-triazol-4-yl)methoxy)phenoxy)acetamido)-4-chlorobenzoic acid (0.05 g, 0.09 mmol, 1 eq.) were dissolved in dimethylformamide (20 mL) and stirred at 22 °C (room temperature) for 10 min. The reaction flask was put under an argon atmosphere and *N*,*N*-diisopropylethylamine (0.04 g, 0.28 mmol, 3 eq.) was slowly added to the reaction mixture *via* syringe. The mixture was then protected from light and further stirred at room temperature. The reaction was monitored by thin layer chromatography (eluent: dichloromethane / methanol, 9:1) and HPLC (eluent: water (+ 0.1% trifluoroacetic acid) / acetonitrile (+ 0.1% trifluoroacetic acid), gradient from 40 to 95% acetonitrile (+ 0.1% trifluoroacetic acid)). The volatiles were evaporated under reduced pressure after full conversion of the starting material. The crude product was purified by flash column chromatography (eluent: dichloromethane / methanol, gradient from 0% methanol to 20% methanol). The purified solid product was dried *in vacuo*. **Yield** 73%, 0.07 g, 0.07 mmol. ^**1**^**H NMR** (300 MHz, DMSO‑*d*_6_) *δ* 14.26 (s, 1H), 8.69 (t, *J* = 5.5 Hz, 1H), 8.63 (d, *J* = 2.2 Hz, 1H), 8.55 (s, 1H), 7.98 (d, *J* = 8.4 Hz, 1H), 7.92 (s, 1H), 7.86 (d, *J* = 7.9 Hz, 1H), 7.43 (d, *J* = 2.7 Hz, 2H), 7.22 (d, *J* = 7.9 Hz, 1H), 7.14–7.01 (m, 3H), 7.00–6.69 (m, 7H), 5.20 (s, 2H), 4.62 (s, 2H), 4.35 (t, *J* = 7.1 Hz, 2H), 3.30 (s, 12H), 1.99 (s, 3H), 1.79 (p, *J* = 7.4 Hz, 2H), 1.50 (q, *J* = 6.9 Hz, 2H), 1.33–1.18 (m, 6H), 0.59 (d, *J* = 8.3 Hz, 6H). ^**13**^**C NMR** (75 MHz, DMSO) *δ* 168.26, 167.68, 166.37, 165.57, 161.88, 153.73, 148.25, 147.84, 147.16, 143.03, 141.12, 140.99, 139.84, 135.27, 134.93, 132.75, 128.89, 126.08, 124.72, 122.44, 121.83, 121.54, 121.39, 118.21, 115.96, 114.97, 114.53, 69.54, 62.56, 49.24, 29.70, 28.81, 25.81, 25.55, 18.83, −1.03, −1.39, −5.48. **HRMS** (ESI) *m/z* calculated for [M]^+^ 926.3823, found 926.3795.

### 2.2 Chemicals and extracts

Commercially available fluorescein and rhodamine fluorophores were purchased from Sigma-Aldrich (rhodamine B: 83689, rhodamine 123: 83702, rhodamine 6G: 83697). 50 mM stock solutions of each fluorophore were prepared in a 94% EtOH and were kept at −20 °C. The reference standard for gallic acid and tannic acid was purchased from Sigma-Aldrich (398,225, 403,040). All additional tannin reference standards (1,2,6-trigalloyl-β-d-glucose, 1,2,3,4,6-pentagalloyl-β-d-glucose, vescalagin, punicalagin, (−)-catechin, (−)-epicatechin, (−)-gallocatechin, (+)-catechin-5-gallate, (−)-catechin-3-gallate, (−)-epicatechin-3-gallate, (−)-epigallocatechin-3-gallate, (−)-gallocatechin-3-gallate), proanthocyanidin A1, A2, B2 and C1) were purchased from Phytolab. The stock solutions for tannin reference compounds were prepared in DMSO (100 mM) and were kept at −20 °C. Tannin containing botanical samples were purchased commercially from Deckel Dyes and included: Aleppo oak gallnuts (*Quercus infectoria*), Sorrel (*Rumex acetosa*), Tara pods (*Caesalpinia spinosa*), Sumac leaf (*Rhus sp.*), Green walnut hulls (*Juglans regia*) and Rhubarb root (*Rheum sp.*). For the extraction of tannins, 5 g of each sample were solubilized in 100 mL of 80% EtOH and were sonicated for 60 min at 60 °C. Ethanol was removed using a Rotary Evaporator (Buchi Rotavapor® R300) at 130 mbar of pressure, with a bath temperature of 45 °C and a cooling temperature of 10 °C for one hour. Next, to remove the water, the EtOH evaporated samples were lyophilized overnight and were reconstituted in 50% EtOH/mQ at a stock concentration of 50 mg mL^−1^.

### 2.3 Spectrofluorometry for the quenching assay

We used the Agilent Cary Eclipse for spectrofluorometric measurements. The voltage of the photomultiplier tube (PMT) was set between 500 and 750 V depending on the fluorophore used. All fluorophores were prepared in 2 mM DMSO stocks and were diluted to the working concentrations (750 nM) in the respective buffer (phosphate-buffered saline, pH = 7.4) in a 3 mL cuvette. The tannic acid standard (Sigma-Aldrich, 403,040) was dissolved in mQ water for both the 100 mM stock solution and the working dilutions. For SiR compounds, the λ_ex_ was set to 650 nm and the emission spectrum was retrieved in the 650–700 nm range. The peak emission at 670 nm was used for quantification. For the calibration curves, at least five data points were used with increasing concentrations of TA. The fluorescence values of all data points were normalized to the blank solution which contained 750 nM of the respective fluorophore and no TA. We applied a linear fit to the natural logarithm of the fluorescence change compared to the blank solution. The absolute fluorescence was expressed in arbitrary units (AU). For the comparison of quenching efficiency between the compounds, the half-maximal inhibitory concentration (IC_50_, the concentration needed to reduce the fluorescent intensity to half of the maximal value), and the maximum quenching effect (E_max_, limiting percentage of fluorescent reduction) was determined. To investigate the effect of different buffers on the quenching efficiency, we prepared a 10× Phosphate-buffer saline (PBS) solution with standard composition: 1.37 M NaCl, 27 mM KCl, 100 mM Na_2_HPO_4_, and 18 mM KH_2_PO_4_. The 1× PBS solution was used for most of the assays unless otherwise stated. The salt components of all buffers were dissolved in mQ.

### 2.4 Spiking experiments in beverage samples

Tomato and cranberry juice samples were purchased from a local supermarket and were kept at 4 °C. During the spectrofluorometric analysis, 1 μL of the unprocessed sample were added to 3 mL of 750 μM of the SiR-5. Fluorescence values were retrieved after reaching equilibrium at 5 min.

### 2.5 Standard wine analytical methods

Red wine bottles were provided by the cellar of Agroscope (Changins). The condensed tannin content of red wine and tannin extract samples were estimated using acid catalyzed hydrolysis (butanolysis) and methylcellulose precipitation (MCP). The acid catalyzed butanolysis was based on a method described by Ribéreau-Gayon & Stonestreet (Ribéreau-Gayon & Stonestreet, 1966). Wine samples were diluted 50 times with mQ water. In a glass tube, 1 mL of the diluted sample was mixed with 3 mL of a hydrolysis reagent that composed of 50% (*v*/v) butanol, 19% (v/v) 6 N HCl, and 150 mg L^−1^ FeSO_4_. The reaction mixture was divided into two 2 mL fractions; one was heated to 100 °C, and the other was kept at room temperature in the dark. After 30 min, the heated tube was cooled down to room temperature, and the absorbance at 550 nm for each fraction was measured with a spectrophotometer (Agilent, Cary 60 UV–Vis). The difference between the two absorbances was multiplied by the dilution factor and the slope of the calibration curve done with leucoanthocyanidol, giving the total tannin concentration in g L^−1^ of leucoanthocyanidol equivalent. The methylcellulose precipitation (MCP) method was done as described in the literature (Sarneckis et al., 2006).

The total anthocyanins content was determined as well using the Puissant-Léon method (Ribéreau-Gayon et al., 2006.). The method was adapted to an autoanalyzer (A25, BioSystem, Barcelona, Spain) and was carried out by adding 380 μL of 1% HCl to 20 μL of sample and by measuring the absorbance at 520 nm after 300 s. Results of the anthocyanin measurements are expressed in mg malvidin-3-*O* glucoside per liter of wine. The total phenolic content was estimated using the Folin–Ciocalteu method (Singleton et al., 1999) adapted to a spectrophotometric autoanalyzer (A25, Bio System, Barcelona, Spain). Results (absorbance at 750 nm corrected by the dilution factor) are expressed as the Folin Index (FI).

### 2.6 Data analysis and statistics

All statistical analysis was done in Python 3.9. The reported data were obtained in at least 3 independent experiments and are represented as mean ± standard deviation unless otherwise indicated. Statistical significance was determined using Mann-Whitney test with the Bonferroni correction where applicable unless otherwise stated (^ns^p > 0.05, *p < 0.05, **p < 0.01, ***p < 0.001, ****p < 0.0001).

## 3 Results

### 3.1 Potency of the TA-induced quenching of rhodamines and their chemical conjugates

Spurred by the observation that TA very strongly quenched the signal of our recently developed calcium-sensing receptors (CaSR) probe containing a silicon-rhodamine moiety (Bátora et al., 2024), we set to explore this effect on rhodamine in general and its utility to quantify tannins. We assessed the potency of the quenching mechanism induced by TA on various rhodamine-derivatives and the structurally similar fluorescein, as depicted in Fig. 1. Specifically, we tested commercially available rhodamine fluorophores (R123, RB, R6G), and five synthesized silicon-rhodamine (SiR) conjugates, including conjugates with only an alkane linker (SiR-1), a PEG linker with a bulky chemical moiety (SiR-2), an alkane linker with a bulky chemical moiety (SiR-3), and two additional conjugates with varying the alkane linker length (SiR-4 and SiR-5).

**Fig. 2:**
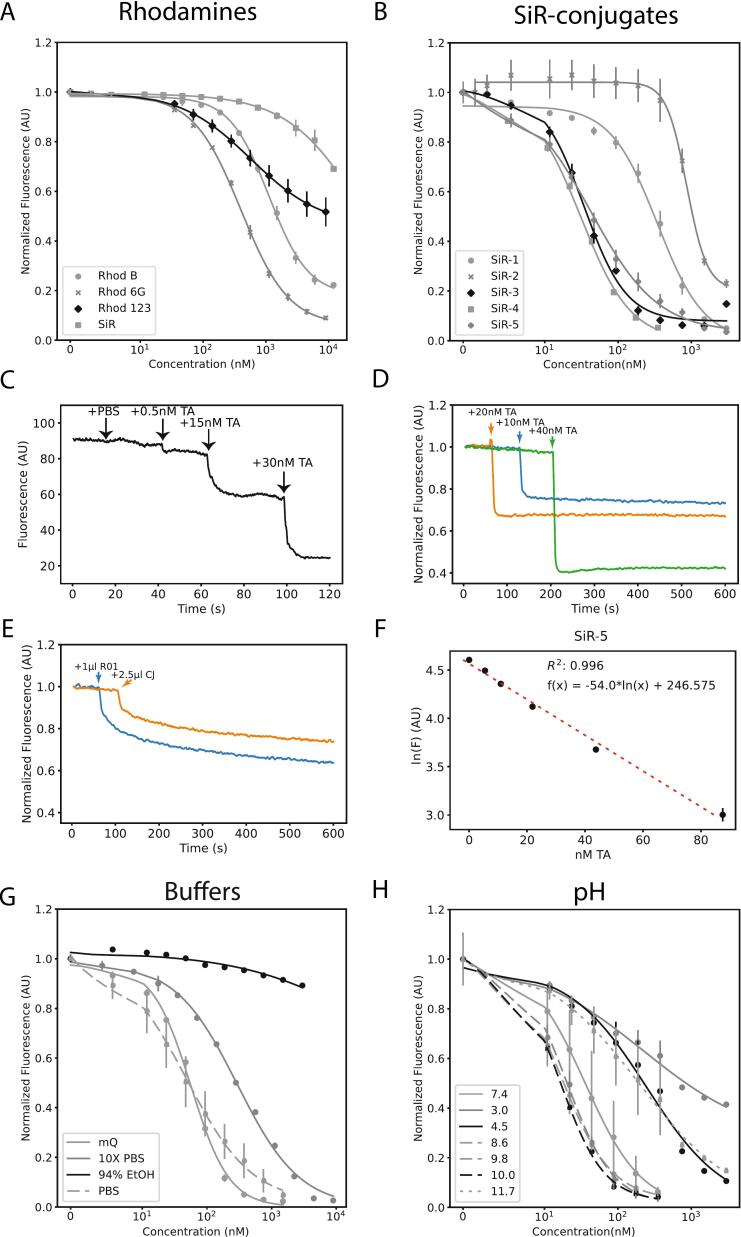
Structure activity relationship (SAR) of rhodamine-based fluorophores and silicon-rhodamine (SiR) conjugates on tannic-acid (TA) induced fluorescence quenching. **A:** Concentration-response curve of the fluorescence quenching of structurally different rhodamine fluorophores with various concentrations of tannic acid (n = 3, in triplicates). **B**: Dose-response curve of SiR-conjugates (*n* = 3–9, in triplicates). **C:** Timelapse plot depicting the absolute fluorescence of 750 nM SiR-5 in response to the addition of various concentrations of tannic acid (TA). The different concentrations of tannic acid were all 2.5 μL in volume and were added sequentially to 3 mL of SiR-5 solution in the cuvette. Solutions were mixed 3 times during the acquisition. **D:** Plot depicting the temporal kinetics of SiR fluorescence quenching reaction. The addition of various concentrations of tannic acid (TA) results in a stable signal up to the length of the measurement (10 min). **E:** Temporal kinetics of SiR fluorescence**.** The addition of red wine and cranberry juice samples results in a sudden drop in the signal that saturates after 5 min. **F:** Calibration curve of SiR-5 to increased concentrations of tannic acid (TA). A linear model was fitted for the concentration range 5 nM–100 nM (*n* = 3, triplicates). **G:** The impact of different buffers on the sensitivity of the quenching mechanism of SiR-5 (n = 3, triplicates). **H:** The impact of varying the pH of mQ on the sensitivity of the quenching mechanism of SiR-5 (n = 3, triplicates).

No quenching effect of TA with fluorescein up was observed up to 3000 nM (Fig. S1). Similarly, SiR was also ineffectively quenched by TA (Fig. 2A). Within the TA concentrations of 5 nM and 9000 nM, the quenching of rhodamine 123 fluorescence had a relatively low E_max_ of 50%, with an IC_50_ value of 578 ± 28.7 nM. The E_max_ and IC_50_ value were 80%, 90% and 1118 ± 51.1 nM, 402 ± 16.2 nM for rhodamine B and rhodamine 6G, respectively (Fig. 2A). Chemical conjugates of SiR comprising of an alkyl linker and a bulky moiety increased the potency of the quenching by an order of magnitude without affecting E_max_, resulting in IC_50_ values of 34.3 ± 2.81 nM 31.3 ± 1.69 nM and 42.5 ± 2.79 nM for SiR-3, SiR-4, and SiR-5, respectively (Fig. 2B). In contrast, SiR conjugates with only the alkyl linker attached (no bulky moiety) did not significantly improve the sensitivity beyond the level of the fluorophore (SiR-1). In addition, a SiR conjugate similar to SiR-3 with a more polar polyethylene glycol (PEG) linker (SiR-2) was not effective in the quenching reaction. Using the highly sensitive SiR-5 conjugate, we measured the limit of detection for TA close to 500 pM (Fig. 2C). The dose-dependent decrease in fluorescence stabilized after 2 s and remained stable up to the 10 min time window measured (Fig. 2D). In contrast, the drop in the fluorescence signal equilibrated after approximately five minutes in cranberry juice and red wine matrices (Fig. 2E). The linearity of the assay with SiR-5 was in the range of 5–100 nM with an R^2^ value of 0.996 (Fig. 2F). The linear range for other rhodamine derivatives were also determined with R^2^ values between 0.993 and 0.999 and are depicted in Fig. S2. The properties of the measured fluorophores are depicted in Table 1.

**Table 1:** Fluorescence quenching of rhodamine fluorophores and related chemical conjugates by tannic acid (TA).

Compound	λ_ex_ (nm)	LOD (nM)	IC_50_ (nM)	LR (nM)	R^2^	E_max_ (%)
Fluorescein	488	–	–	-	-	–
SiR	650	-	-	-	-	-
Rhodamine B	545	90	1117 ± 51.2	94–3000	0.993	80
Rhodamine 123	505	20	578 ± 28.7	-	-	50
Rhodamine 6G	525	50	402 ± 16.1	35–563	0.999	90
SiR-1	650	20	342 ± 41.7	12–750	0.999	100
SiR-2	650	120	340 ± 26.9	12–750	1	100
SiR-3	650	0.5	34.3 ± 2.80	5–100	1	95
SiR-4	650	0.5	31.3 ± 1.69	5–100	0.998	95
SiR-5	650	0.5	42.5 ± 2.79	5–100	0.996	100

*Abbreviations: λ*_*ex*_*, Excitation wavelength; E*_*max*_*, maximum efficacy; LOD, Limit of Detection; LR, linear range; IC*_*50*_*, half-maximal inhibitory concentration; SiR, silicon-rhodamine; R*^*2*^*, coefficient of determination.*

### 3.2 Buffer and pH dependence of the SiR-5 quenching mechanism

**Fig. 3:**
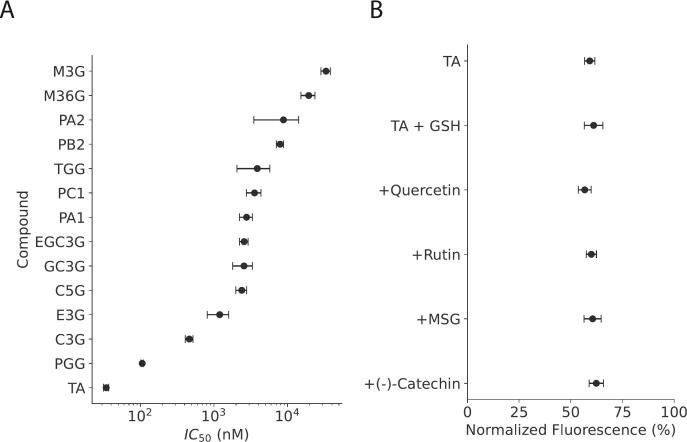
Selectivity of SiR-5 towards structurally diverse tannins, anthocyanins and miscellaneous food constituents. A: The selectivity of SiR-5 was assessed on a structurally diverse panel of tannins. In addition, the selectivity towards two anthocyanins abundant in red wine, malvidin-3-*O*-glucoside (M3G) and malvidin-3-*O*-(6-*O*-p-coumaroyl)-glucoside (M36CG) was also measured. Plot depicts the IC_50_ value of the quenching reaction for each reference compound in the logarithmic scale (n = 3, in triplicates). No quenching was observed for the selected ellagitannins (vescalagin, punicalagin), (−)-catechin, (−)-gallocatechin, (−)-epicatechin and gallic acid (GA). **B:** The addition of common polyphenols and other food ingredients does not interfere with the signal intensity. The compounds were administered in the presence of 15 nM TA and were compared with the absolute fluorescence intensity of 750 nM of SiR-5. (n = 3, in triplicates). Abbreviations: TGG, 1,2,6-trigalloyl-β-d-glucose; PGG, 1,2,3,4,6-pentagalloyl-β-d-glucose;C5G, (+)-catechin-5-gallate; C3G,(−)-catechin-3-gallate; E3G, (−)-epicatechin-3-gallate; EGC3G, (−)-epigallocatechin-3-gallate; GC3G, (−)-gallocatechin-3-gallate);GSH, glutathione; PA1, proanthocyanidin A1; PA2 proanthocyanidin A2; PB2, proanthocyanidin B2; PC1, proanthocyanidin C1; TA, tannic acid; M3G, malvidin-3-O glucoside; M36CG, malvidin-3-O-(6-O-p-coumaroyl) glucoside; MSG, monosodium glutamate

As previous reports on fluorescence quenching based TA probes have reported a strong pH-dependence (Ahmed et al., 2015), we evaluated the effect of different buffers and pH on the sensitivity of SiR-5 quenching. We found that the mechanism was most potent in aqueous buffers with balanced ionic strength, as we observed no quenching or a diminished potency in 94% ethanol and 10× PBS, respectively (Fig. 2G). We found no statistically significant difference in the IC_50_ values between PBS and mQ water. Next, we evaluated the effect of pH on the potency of the quenching mechanism in mQ. We found that both highly acidic (3.0–4.5) and highly basic pH (11.7) significantly diminished the potency of the quenching mechanism (IC_50_ = 217.6 ± 108.9 nM, 247.3 ± 85 nM, 195.0 + 49.4 nM for pH = 3.0, 4.5 and 11.7, respectively) induced by TA (Fig. 3H). The optimal pH for achieving the highest possible potency was found to be in the slightly basic range between 7.4 and 10.0 (IC_50_ = 36.0 ± 2.0 nM, 17.4 ± 0.45 nM, 20.0 ± 1.15 nM and 16.0 ± 0.67 nM for pH = 7.4, 8.6, 9.8 and 10.0, respectively). To minimize the impact of pH, we used PBS pH = 7.4 for the rest of the study.

### 3.3 Characterization of the selectivity of the quenching mechanism on chemically diverse tannins

The selectivity of the SiR-5 quenching mechanism was assessed on a panel of 20 structurally diverse tannins and related polyphenols that included gallotannins (gallic acid, 1,2,6-trigalloyl-β-d-glucose, 1,2,3,4,6-pentagalloyl-β-d-glucose, TA) and ellagitannins (vescalagin, punicalagin), flavan-3-ols ((−)-catechin, (−)-epicatechin, (−)-gallocatechin, (+)-catechin-5-gallate, (−)-catechin-3-gallate, (−)-epicatechin-3-gallate, (−)-epigallocatechin-3-gallate, (−)-gallocatechin-3-gallate), proanthocyanidins (proanthocyanidin A1, A2, B2, C1), plus two structurally closely related malvidins (malvidin-3-*O-*glucoside, malvidin 3-*O*-(6-*O*-p-coumaroyl)-glucoside). The results are depicted in Table 2. The quenching was especially sensitive towards the hydrolysable tannins 1,2,3,4,6-pentagalloyl-β-d-glucose (PGG), TA and (−)-catechin-3-gallate (C3G) with IC_50_ values of 34.31 ± 2.81, 105.94 ± 3.74 and 303.85 ± 127.42 nM, respectively (Fig. 3A, Table 2, Fig. S3). The assay was >100 fold and ∼ 1000 fold more selective for TA over the selected proanthocyanidins and malvidins, respectively. Importantly, no quenching interaction was observed for the measured ellagitannins (vescalagin, punicalagin), catechin, and epicatechin monomers (Fig. 3B, Fig. S3).

**Table 2:** Selectivity of the quenching effect towards structurally different tannins, flavan-3ols and anthocyanins.

Compound	IC_50_ (nM ± STD)	Compound	IC_50_ (nM ± STD)
TA	34 ± 2	PB2	7942 ± 859
PGG	105 ± 3	M36G	19,427 ± 4188
C3G	462 ± 56	M3G	33,369 ± 4961
E3G	1197 ± 386	Punicalagin	N/A
C5G	2389 ± 399	Vescalagin	N/A
GC3G	2563 ± 773	GA	N/A
EGC3G	2571 ± 345	EC	N/A
PA1	2618 ± 494	GC	N/A
TGG	3882 ± 1836	C	N/A
PA2	7370 ± 3072	PC1	N/A

Abbreviations: TGG, 1,2,6-trigalloyl-β-d-glucose; PGG, 1,2,3,4,6-pentagalloyl-β-d-glucose;C5G, (+)-catechin-5-gallate; C3G,(−)-catechin-3-gallate; E3G, (−)-epicatechin-3-gallate; EGC3G, (−)-epigallocatechin-3-gallate; GC3G, (−)-gallocatechin-3-gallate); PA1, proanthocyanidin A1; PA2 proanthocyanidin A2; PB2, proanthocyanidin B2; PC1,proanthocyanidin C1; TA, tannic acid; M3G, malvidin-3-O-glucoside; M36G, malvidin-3-O-(6-O-p-coumaroyl) glucoside.

### 3.4 Quantification and spiking of TA in unprocessed beverage samples

To elucidate potential matrix effects, we quantified the TA concentration in tomato juice (TJ) and cranberry juice (CJ) samples. We found 23.92 ± 1.17 nM and 11.34 ± 2.58 nM of TA in TJ and CJ, respectively (Table 3). We spiked the samples with TA concentrations between 5.85 nM and 23.44 nM, which we could reliably quantify within 20% deviations from the real value for both samples (Table 3).

**Table 3:** Quantification of tannic acid (TA) and spiking experiments in tomato juice and cranberry juice samples.

Sample ID	Spiked (nM)	Found (nM)	Recovery (%)	RSD (%)
TJ (*n* = 3)	0	23.92 ± 1.17	–	–
	5.85	30.41 ± 0.38	110.72	23.80
	23.44	44.37 ± 2.43	87.22	13.17
CJ (n = 3)	0	11.34 ± 2.57		–
	5.85	17.13 ± 2.52	98.76	29.17
	11.72	24.79 ± 2.28	114.74	10.79

Abbreviations: RSD: relative standard deviation.

### 3.5 Comparison of SiR-quenching with common tannin quantification methods in red wine

**Fig. 4:**
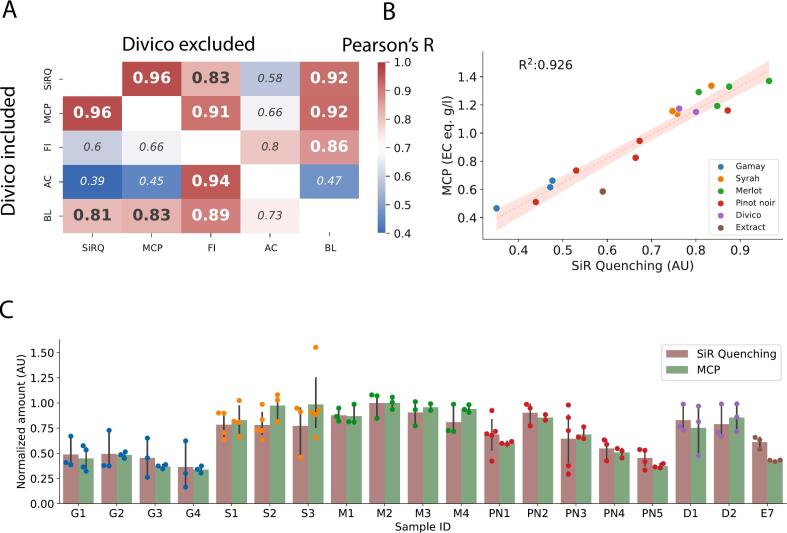
Comparison and validation of SiR-5 quenching with methylcellulose precipitation (MCP) in the analysis of red wines and a grape extract. (For interpretation of the references to color in this figure legend, the reader is referred to the web version of this article.) **A:** Heatmap depicting the Pearson's correlation coefficient (R) for all methods by which the red wine samples were quantified. The upper heatmap shows the correlations of the Divico excluded matrix, whereas the lower heatmap shows the correlations of the Divico included matrix. **B:** Linear regression analysis of the methylcellulose precipitation method (MCP) and the quenching method (SiR-5 quenching). The red line indicates the linear fit, whereas the dimmer halo represents the 95% confidence interval. The coefficient of determination (R^2^) is depicted in the top left corner (*p* < 0.0001). **C:** The bar plot shows the comparison of the variability between the methylcellulose precipitation (MCP) method and the SiR quenching. Within the two methods, each value was normalized to the highest value in the group to enable the direct comparison. No significant difference was observed for any of the samples (*p* > 0.1 for all samples, Mann-Whitney test). Abbreviations: SiRQ, SiR-5-quenching; MCP, methylcellulose precipitation; FI, Folin-Ciocalteu index; AC, anthocyanin content; BL, butanolysis

Because the selectivity data indicated strong quenching of SiR-5 by catechin-gallates and to a lesser extent by condensed tannins, which both are common in red wine, we next pursued to validate and test the robustness of the SiR-quenching method with real world red wine samples. For the analysis, 18 red wine samples made from various grape varieties covering a wide spectrum of tannin and anthocyanin levels (Gamay, Pinot noir, Syrah, Merlot, Divico) and a grape skin extract were selected (Table 4). In addition to SiR-quenching, we quantified the total phenolic content (Folin-Ciocalteu), the anthocyanin content, and the condensed tannin content of the samples. Two established methods were used for the quantification of condensed tannins: a colorimetric approach measuring the released monomers of condensed tannins after acidic hydrolyses (Butanolysis) and a precipitation-based assay (methylcellulose precipitation). The results of the Folin-Ciocalteu index (FI) and the butanolysis (BL) was strongly influenced by the anthocyanin content, especially beyond 1000 mg L^−1^ (Pearson's *R* = 0.94 (*p* < 0.0001) and 0.73 (*p* = 0.0003) for FI and BL, respectively). Thus, we conducted the correlation analysis with the inclusion and the exclusion of the Divico samples (anthocyanin content >1500 mg L^−1^). The highest correlation was observed between the methylcellulose precipitation (MCP) and the SiR-5 quenching (Pearson's *R* = 0.96, p < 0.0001), which was not influenced by the high anthocyanin content (>1500 mg L^−1^) of the Divico samples (Fig. 4A). The SiR-5-quenching assay also showed a high correlation with the butanolysis and the FI when the Divico samples were excluded (Pearson's *R* = 0.92 and 0.83, respectively, p < 0.0001 for both). The values of the MCP also highly correlated with those of the butanolysis and of the FI, which was again strongly dependent on the inclusion of the Divico samples. The regression analysis with the coefficients of determination values for the SiR-5-quenching and MCP is depicted in Fig. 4B, whereas the rest of the comparisons are depicted in Fig. S4 and Fig. S5. The variability between the individual measurement points were comparable for SiR-quenching and MCP for all samples measured and are shown in Fig. 4C.

**Table 4:** Comparison of existing methods with SiR Quenching on the quantification of tannins for real world samples.

Sample ID	Species	Variety/Type	Year	Barrel	SiR Quenching	MCP	FI	Anthocyanin	Butanolysis
G1	*Vitis vinifera*	Gamay	2022	steel	0.47	0.62	30.9	367	0.89
G2	*Vitis vinifera*	Gamay	2022	steel	0.48	0.66	29.7	377	0.81
G3	*Vitis vinifera*	Gamay	2022	steel	0.44	0.51	28.3	363	0.75
G4	*Vitis vinifera*	Gamay	2017	steel	0.35	0.47	26	113	0.9
S1	*Vitis vinifera*	Syrah	2022	steel	0.76	1.14	41.6	592	1.71
S2	*Vitis vinifera*	Syrah	2022	steel	0.84	1.34	43.6	576	1.98
S3	*Vitis vinifera*	Syrah	2022	steel	0.75	1.16	44	592	1.79
M1	*Vitis vinifera*	Merlot	2022	steel	0.85	1.19	43.1	447	2.39
M2	*Vitis vinifera*	Merlot	2022	steel	0.97	1.37	45.4	472	2.25
M3	*Vitis vinifera*	Merlot	2022	steel	0.88	1.33	45	479	2.54
M4	*Vitis vinifera*	Merlot	2017	steel	0.81	1.29	42.9	194	2.48
PN1	*Vitis vinifera*	Pinot noir	2022	steel	0.66	0.82	34.3	297	1.76
PN2	*Vitis vinifera*	Pinot noir	2022	steel	0.87	1.16	41.8	354	2.16
PN3	*Vitis vinifera*	Pinot noir	2022	steel	0.67	0.94	38.6	407	1.83
PN4	*Vitis vinifera*	Pinot noir	2022	steel	0.53	0.73	27.9	198	0.95
PN5	*Vitis vinifera*	Pinot noir	2017	steel	0.44	0.51	25.2	78	1.21
D1	*Interspecific hybrid*	Divico	2021	steel	0.80	1.15	77.6	1672	3.43
D2	*Interspecific hybrid*	Divico	2021	oak	0.76	1.17	80.8	1680	3.07
E1	*Rhus coriaria*	Extract	N/A	N/A	0.49	1.42	405	0	0.23
E2	*Rumex acetosa*	Extract	N/A	N/A	0.12	0.10	79.2	51	1.54
E3	*Quercus infectoria*	Extract	N/A	N/A	0.69	2.27	1440	0	0.09
E4	*Cesalpina spinosa*	Extract	N/A	N/A	0.07	1.37	1265	0	0.57
E5	*Juglans regia*	Extract	N/A	N/A	0.09	0.09	204	649	1.82
E7	*Reum sinensis*	Extract	N/A	N/A	0.31	0.20	379	774	5.26
E7	*Vitis vinifera*	Extract	N/A	N/A	0.59	0.59	14.2	4	0.89

SiR Quenching is expressed in arbitrary units. MCP is expressed in Epicatechin equivalents (g L^−1^), anthocyanins in mg L^−1^ and butanolysis in g L^−1^ leucoanthocyanidol equivalents.

Abbreviations: MCP, methylcellulose precipitation; FI, Folin-Ciocalteu Index.

### 3.6 Comparison of SiR-quenching with common tannin quantification methods in tannin extracts

We assessed the adaptability and accuracy of SiR-5-quenching and other tannin quantification methods in six tannin extracts from diverse botanical origins. We found the strongest correlation (Pearson's *R* = 0.86) between the MCP method and the FI, followed by the anthocyanin content and the butanolysis (Pearson's *R* = 0.85) and the SiR-quenching and the MCP (Pearson's *R* = 0.72) (Fig. S6). As shown in Table 4, the divergence between the SiR-quenching (0.07) and the MCP (1.37) on the *Caesalpinia spinosa* extract (Tara pods) led to the reduced correlation between the two methods. No correlation was observed between the butanolysis and the rest of the methods.

### 3.7 Discussion of results

The sensitive and selective quantification of tannins or subsets of tannins that are representative markers is necessary to gain insights on their sensory attributes, stability, and potential health hazards in foodstuff and beverages (Nghia et al., 2023). To this end, we developed a novel, single-component assay that is based on the fluorescence quenching of specific silicon rhodamine conjugates. The two major findings of the study are a) the in-depth characterization of the novel assay using TA and b) the comparison and validation of the assay in estimating astringency in red wine with standard methods.

We report differential sensitivity of the quenching mechanism using TA, which strongly depended on the structure of the rhodamine fluorophore and the chemical conjugation. Attachment of a bulky chemical moiety to the SiR fluorophore, and the use of apolar, alkyl linkers for conjugation (SiR-3, SiR-4, and SiR-5) yielded the highest potency of the quenching mechanism. The length of the linker did not influence the quenching reaction (SiR-4 and SiR-5), whereas the introduction of a PEG linker (SiR-2) resulted in an order of magnitude decrease in potency. The approximately two orders of magnitude wide linear range with SiR-5 was comparable to recently described assays (Ahmed et al., 2015; Arul et al., 2023; Li et al., 2015; Liu et al., 2019; Nghia et al., 2023; Wang et al., 2024), and was spanning the low nanomolar range (5–100 nM). The comparison of our assay with recently developed methods is depicted in Table S1. Taken together, our finding that specific chemical conjugates drastically improved the assay sensitivity may also allow the optimization of the selectivity of the SiR-quenching assay towards specific tannins of interest in the future.

Our results unveil an intriguing substochiometic mechanism. For hydrolysable gallotannins, the potency of the quenching mechanism (IC_50_ value) was quasi-logarithmically proportional to the number of gallic acid moieties in the molecules (Fig. 3, Table 2, Fig. S3), whereas gallic acid did not show any observable quenching reaction below 3 μM. For condensed tannins and flavan-3-ol gallates, the polymerization degree and specific gallic acid configurations determined the quenching potency (C3G), respectively, whereas ellagitannins and flavan-3-ols without gallic acid esters had no effect, and the degree of catechin polymerization reduced the quenching potency. Our results are in agreement with previous reports that proposed such tannin-induced quenching to be driven by formation of tannin-dye complexes through hydrogen bonding and π-π stacking (Chen et al., 2022; Pucci et al., 2022; Wang et al., 2024). However, more research is needed in this direction to fully uncover the molecular interactions of these complexes with rhodamine fluorophores.

The adaptation of novel quantification assays to practical applications necessitates the comparison of the new method with standard techniques. We tested the robustness of the SiR-5 assay by comparing the readout on 18 red wine samples from five distinct grape varieties and ages with two distinct methods for condensed tannin quantification used in enology: butanolysis and MCP. Even though the selectivity of precipitation-based methods like the Harbertson-Adams assay or the MCP towards specific tannin molecules is not fully elucidated, the methods are preferred and readily used in the industry as they resemble and serve as good predictors (R^2^ = 0.47–0.90) of astringency, which is the major goal of condensed tannin quantification in winemaking (Pavez et al., 2022; Wilhelmy et al., 2021). Conversely, it is difficult to scale such precipitation-based assays to multi-well settings as the absorbance-based readout requires the separation of the supernatant from the pellet. Our data unveiled a strong linearity between our method and MCP (R^2^ = 0.92), suggesting that even though SiR-5 quenching might differ in the selectivity profile than the MCP method and mainly quantifies C3G, it is a comparable marker for astringency in red wine as the MCP method. The limitation of differential sensitivity was highlighted when the two methods were compared on the quantification of tannin extracts from various plant taxa (Fig. S6) with distinct molecular compositions, showing substantially lower R^2^ values (0.72). Notably, the correlation between the MCP and butanolysis was also diminished (R^2^ = −0.68 in tannin extracts *vs* R^2^ = 0.83 in red wine), indicating the need of separate and careful method validation for each species. Nevertheless, the high linearity between SiR-5 quenching and MCP in red wine with distinct grape varieties and ages suggests that the assay might work robustly for the Vitis genus.

As highlighted by our analysis, in the quantification of wine tannins, butanolysis exhibits a high interference with anthocyanins. In traditional red grape varieties, the total polyphenols measured with FI also gives an accurate estimation of tannin content, because the tannin concentration is 10 times higher than the concentration of all the other antioxidants. Thus, the correlations between quenching and FI or MCP and FI were high (R^2^ = 0.83 and 0.91 respectively). Although both of these methods are scalable and have been shown to also highly correlate with astringency (R^2^ = 0.8) (Rinaldi et al., 2012), such interference may limit the accuracy of the quantification in resistant red grape varieties (PIWI), that are introduced to the market as means to decrease the use of pesticides on the vineyard in compliance with environmental goals (Duley et al., 2023). Wines produced from these varieties are generally highly colored, with the anthocyanin concentration reaching 2000–3000 mg L^−1^ (He et al., 2012). Therefore, when the concentration of anthocyanins is in a similar range to tannins, such as PIWI grapes like Divico, methods like the FI and butanolysis are less reliable in the estimation of tannins.

## 4 Conclusions

We report a novel quantitative assay for specific tannins using a quenching mechanism of rhodamine derivatives with a tunable sensitivity in the range of 5 nM to 1000 nM. The assay can be used for the accurate quantification of TA and C3G in foodstuff, and as a marker for astringency in red wine with a robust correlation with the MCP method requiring only 1 μL of sample volume. Together, the high sensitivity and selectivity profile of this single-component method permit its adaptation for on-site analysis.

## Funding sources

This study was funded by the University of Bern and the SNFS Sinergia grant 10000189

## CRediT authorship contribution statement

**Daniel Bátora:** Writing – original draft, Visualization, Software, Methodology, Investigation, Formal analysis, Conceptualization. **Ágnes Dienes-Nagy:** Writing – review & editing, Validation, Methodology, Conceptualization. **Liming Zeng:** Writing – review & editing, Validation, Methodology, Conceptualization. **Christian E. Gerber:** Writing – review & editing, Methodology. **Jérôme P. Fischer:** Writing – review & editing, Conceptualization. **Martin Lochner:** Writing – review & editing, Resources, Methodology. **Jürg Gertsch:** Writing – review & editing, Supervision, Conceptualization, Project administration.

## Declaration of competing interest

The authors declare that they have no known competing financial interests or personal relationships that could have appeared to influence the work reported in this paper.

## Data Availability

All raw data associated to this article will be made available on request.

## Appendix A Supplementary data

Supplementary material Image 1

## References

[bb0005] Ahmed G.H.G., Laíño R.B., Calzón J.A.G., García M.E.D. (2015). Fluorescent carbon nanodots for sensitive and selective detection of tannic acid in wines. Talanta.

[bb0010] Andrade R.G., Ginani J.S., Lopes G.K.B., Dutra F., Alonso A., Hermes-Lima M. (2006). Tannic acid inhibits in vitro iron-dependent free radical formation. Biochimie.

[bb0015] Arul P., Nandhini C., Huang S.-T., Gowthaman N.S.K. (2023). Development of water-dispersible Dy(III)-based organic framework as a fluorescent and electrochemical probe for quantitative detection of tannic acid in real alcoholic and fruit beverages. Analytica Chimica Acta.

[bb0020] Bátora D., Fischer J.P., Kaderli R.M., Varga M., Lochner M., Gertsch J. (2024).

[bb0025] Bigham A., Rahimkhoei V., Abasian P., Delfi M., Naderi J., Ghomi M., Dabbagh Moghaddam F., Waqar T., Nuri Ertas Y., Sharifi S., Rabiee N., Ersoy S., Maleki A., Nazarzadeh Zare E., Sharifi E., Jabbari E., Makvandi P., Akbari A. (2022). Advances in tannic acid-incorporated biomaterials: Infection treatment, regenerative medicine, cancer therapy, and biosensing. Chemical Engineering Journal.

[bb0030] Cabral-Hipólito N., Molina-Ramírez B.S., Castillo-Maldonado I., Meza-Velázquez R., García-Garza R., Gauna S.-E.V., Pedroza-Escobar D. (2022). Tannic acid exhibits adjuvant activity by enhancing humoral and cell-mediated immunity against BSA as a protein antigen. Protein and Peptide Letters.

[bb0035] Chen C., Yang H., Yang X., Ma Q. (2022). Tannic acid: A crosslinker leading to versatile functional polymeric networks: A review. RSC Advances.

[bb0040] Chen Z., Zhang X., Cao H., Huang Y. (2013). Chitosan-capped silver nanoparticles as a highly selective colorimetric probe for visual detection of aromatic ortho-trihydroxy phenols. Analyst.

[bb0045] Chung K.T., Wong T.Y., Wei C.I., Huang Y.W., Lin Y. (1998). Tannins and human health: A review. Critical Reviews in Food Science and Nutrition.

[bb0050] Dahlin J.L., Auld D.S., Rothenaigner I., Haney S., Sexton J.Z., Nissink J.W.M., Wagner B.K. (2021). Nuisance compounds in cellular assays. Cell Chemical Biology.

[bb0055] Das A.K., Islam M.N., Faruk M.O., Ashaduzzaman M., Dungani R. (2020). Review on tannins: Extraction processes, applications and possibilities. South African Journal of Botany.

[bb0060] Degano I., Mattonai M., Sabatini F., Colombini M.P. (2019). A mass spectrometric study on tannin degradation within dyed woolen yarns. Molecules.

[bb0065] Duley G., Ceci A.T., Longo E., Boselli E. (2023). Oenological potential of wines produced from disease-resistant grape cultivars. Comprehensive Reviews in Food Science and Food Safety.

[bb0070] Farha A.K., Yang Q.-Q., Kim G., Li H.-B., Zhu F., Liu H.-Y., Corke H. (2020). Tannins as an alternative to antibiotics. Food Bioscience.

[bb0075] Grossenbacher P., Essers M.C., Moser J., Singer S.A., Häusler S., Stieger B., Lochner M. (2022). Bioorthogonal site-selective conjugation of fluorescent dyes to antibodies: Method and potential applications. RSC Advances.

[bb0080] Hara K., Ohara M., Hayashi I., Hino T., Nishimura R., Iwasaki Y., Ogawa T., Ohyama Y., Sugiyama M., Amano H. (2012). The green tea polyphenol (−)-epigallocatechin gallate precipitates salivary proteins including alpha-amylase: Biochemical implications for oral health. European Journal of Oral Sciences.

[bb0085] Harbertson J.F., Hodgins R.E., Thurston L.N., Schaffer L.J., Reid M.S., Landon J.L., Adams D.O. (2008). Variability of tannin concentration in red wines. American Journal of Enology and Viticulture.

[bb0090] He F., Liang N.-N., Mu L., Pan Q.-H., Wang J., Reeves M.J., Duan C.-Q. (2012). Anthocyanins and their variation in red wines I. Monomeric anthocyanins and their color expression. Molecules.

[bb0095] Li G.-W., Hong L., Tong M.-S., Deng H.-H., Xia X.-H., Chen W. (2015). Determination of tannic acid based on luminol chemiluminescence catalyzed by cupric oxide nanoparticles. Analytical Methods.

[bb0100] Liao Y., Sun Y., Peng X., Qi B., Li Y. (2023). Effects of tannic acid on the physical stability, interfacial properties, and protein/lipid co-oxidation characteristics of oil body emulsions. Food Hydrocolloids.

[bb0105] Liu X., Zhang W., Yang C., Yao Y., Huang L., Li S., Wang J., Ji Y. (2019). Rapid and selective fluorometric determination of tannic acid using MoO3-x quantum dots. Microchimica Acta.

[bb0110] Ma J., Zhou Z., Li K., Li K., Liu L., Zhang W., Xu J., Tu X., Du L., Zhang H. (2021). Novel edible coating based on shellac and tannic acid for prolonging postharvest shelf life and improving overall quality of mango. Food Chemistry.

[bb0115] Nghia N.N., Huy B.T., Khanh D.N.N., van Cuong N., Li H., Lee Y.-I. (2023). Food Chemistry.

[bb0120] Ozhathil L.C., Delalande C., Bianchi B., Nemeth G., Kappel S., Thomet U., Abriel H. (2018). Identification of potent and selective small molecule inhibitors of the cation channel TRPM4. British Journal of Pharmacology.

[bb0125] Panzella L., Napolitano A. (2022). Condensed tannins, a viable solution to meet the need for sustainable and effective multifunctionality in food packaging: Structure, sources, and properties. Journal of Agricultural and Food Chemistry.

[bb0130] Pavez C., González-Muñoz B., O’Brien J.A., Laurie V.F., Osorio F., Núñez E., Brossard N. (2022). Red wine astringency: Correlations between chemical and sensory features. LWT.

[bb0135] Picariello L., Rinaldi A., Forino M., Errichiello F., Moio L., Gambuti A. (2020). Effect of different enological tannins on oxygen consumption, phenolic compounds, color and astringency evolution of Aglianico wine. Molecules.

[bb0140] Pucci C., Martinelli C., de Pasquale D., Battaglini M., Di Leo N., Degl'Innocenti A., Belenli Gümüş M., Drago F., Ciofani G. (2022). Tannic acid-Iron complex-based nanoparticles as a novel tool against oxidative stress. ACS Applied Materials & Interfaces.

[bb0145] Ribéreau-Gayon P., Glories Y., Maujean A., Dubourdieu D. (2006). https://www.wiley.com/en-us/Handbook+of+Enology%2C+Volume+2%3A+The+Chemistry+of+Wine+-%C2%A0Stabilization+and+Treatments%2C+2nd+Edition-p-9780470010389.

[bb0150] Ribéreau-Gayon P., Stonestreet E. (1966). Dosage des tanins du vin rouge et determination de leur structure. Chim Anal.

[bb0155] Rinaldi A., Gambuti A., Moio L. (2012). Application of the SPI (saliva precipitation index) to the evaluation of red wine astringency. Food Chemistry.

[bb0160] Rouxinol M.I., Martins M.R., Salgueiro V., Costa M.J., Barroso J.M., Rato A.E. (2023). Climate effect on morphological traits and polyphenolic composition of red wine grapes of Vitis vinifera. Beverages.

[bb0165] Samtiya M., Aluko R.E., Dhewa T. (2020). Plant food anti-nutritional factors and their reduction strategies: An overview. Food Production, Processing and Nutrition.

[bb0170] Sarneckis C.J., Dambergs R.G., Jones P., Mercurio M., Herderich M.J., Smith P.A. (2006). Quantification of condensed tannins by precipitation with methyl cellulose: Development and validation of an optimised tool for grape and wine analysis. Australian Journal of Grape and Wine Research.

[bb0175] Singleton V.L., Orthofer R., Lamuela-Raventós R.M., Packer L. (1999). Methods in enzymology oxidants and antioxidants part a.

[bb0180] Soyocak A., Kurt H., Cosan D.T., Saydam F., Calis I.U., Kolac U.K., Gunes H.V. (2019). Tannic acid exhibits anti-inflammatory effects on formalin-induced paw edema model of inflammation in rats. Human & Experimental Toxicology.

[bb0185] Tu Q., Liu S., Li Y., Zhang L., Wang Z., Yuan C. (2022). The effects of regions and the wine aging periods on the condensed tannin profiles and the astringency perceptions of cabernet sauvignon wines. Food Chemistry: X.

[bb0190] Wang J.-D., Zhao Y., Ghorai S.K., Li Z., Weng Y.-H., Cao S.-H., Li Y.-Q. (2024). Derivative-synchronous fluorescence spectroscopy enhanced surface plasmon coupled emission for sensitive detection of tannic acid. Sensors and Actuators B: Chemical.

[bb0195] Wilhelmy C., Pavez C., Bordeu E., Brossard N. (2021). A review of tannin determination methods using spectrophotometric detection in red wines and their ability to predict astringency. South African Journal of Enology and Viticulture.

[bb0200] Zhang L., Guan Q., Jiang J., Khan M.S. (2023). Tannin complexation with metal ions and its implication on human health, environment and industry: An overview. International Journal of Biological Macromolecules.

