# Supplementary material for: Hypersensitive quantification of major astringency markers in food and wine by substoichiometric quenching of silicon-rhodamine conjugates
Source: Food Chem X. 2024 Jun 25;23:101592. doi: 10.1016/j.fochx.2024.101592 (PMC11261284; doi:10.1016/j.fochx.2024.101592)
Supplement: Supplementary file 1 — Supplementary material [file mmc1.docx]

Ahmed, G.H.G., Laíño, R.B., Calzón, J.A.G., & García, M.E.D. (2015). Fluorescent carbon nanodots for sensitive and selective detection of tannic acid in wines. Talanta, 132, 252–257.

Arul, P., Nandhini, C., Huang, S.-T., & Gowthaman, N.S.K. (2023). Development of water-dispersible Dy(III)-based organic framework as a fluorescent and electrochemical probe for quantitative detection of tannic acid in real alcoholic and fruit beverages. *Analytica Chimica Acta*, *1274*, 341582.

Li, G.-W., Hong, L., Tong, M.-S., Deng, H.-H., Xia, X.-H., & Chen, W. (2015). Determination of tannic acid based on luminol chemiluminescence catalyzed by cupric oxide nanoparticles. Analytical Methods, 7, 1924–1928.

Liu, X., Zhang, W., Yang, C., Yao, Y., Huang, L., Li, S., … Ji, Y. (2019). Rapid and selective fluorometric determination of tannic acid using MoO3-x quantum dots. Microchimica Acta, 186, 247.

Nghia, N.N., Huy, B. T.B.T., Khanh, D.N.N., van Cuong, N., Li, H., & Lee, Y.-I. (2023). Food Chemistry, 410, 135466.
